# Correlation of Feline Coronavirus Shedding in Feces with Coronavirus Antibody Titer

**DOI:** 10.3390/pathogens9080598

**Published:** 2020-07-22

**Authors:** Sandra Felten, Ute Klein-Richers, Regina Hofmann-Lehmann, Michèle Bergmann, Stefan Unterer, Christian M. Leutenegger, Katrin Hartmann

**Affiliations:** 1 Clinic of Small Animal Medicine, Centre for Clinical Veterinary Medicine, LMU Munich, Veterinaerstrasse 13, 80539 Munich, Germany; u.klein@medizinische-kleintierklinik.de (U.K.-R.); n.bergmann@medizinische-kleintierklinik.de (M.B.); s.unterer@medizinische-kleintierklinik.de (S.U.); hartmann@lmu.de (K.H.); 2 Clinical Laboratory, Vetsuisse Faculty, Department of Clinical Diagnostics and Services, and Center for Clinical Studies, University of Zurich, Winterthurerstrasse 260, 8057 Zurich, Switzerland; rhofmann@vetclinics.uzh.ch; 3 IDEXX Laboratories, Inc., 2825 KOVR Dr., West Sacramento, CA 95605, USA; Christian.Leutenegger@antechmail.com

**Keywords:** feline infectious peritonitis (FIP), feline coronavirus (FCoV), carrier, feline enteric coronavirus (FECV), serology, transmission, multi-cat household

## Abstract

Background: Feline coronavirus (FCoV) infection is ubiquitous in multi-cat households. Responsible for the continuous presence are cats that are chronically shedding a high load of FCoV. The aim of the study was to determine a possible correlation between FCoV antibody titer and frequency and load of fecal FCoV shedding in cats from catteries. Methods: Four fecal samples from each of 82 cats originating from 19 German catteries were examined for FCoV viral loads by quantitative reverse transcriptase polymerase chain reaction (RT-qPCR). Additionally, antibody titers were determined by an immunofluorescence assay. Results: Cats with antibodies were more likely to be FCoV shedders than non-shedders, and there was a weak positive correlation between antibody titer and mean fecal virus load (Spearman *r* = 0.2984; *p* = 0.0072). Antibody titers were significantly higher if cats shed FCoV more frequently throughout the study period (*p* = 0.0063). When analyzing only FCoV shedders, cats that were RT-qPCR-positive in all four samples had significantly higher antibody titers (*p* = 0.0014) and significantly higher mean fecal virus loads (*p* = 0.0475) than cats that were RT-qPCR-positive in only one, two, or three samples. Conclusions: The cats’ antibody titers correlate with the likelihood and frequency of FCoV shedding and fecal virus load. Chronic shedders have higher antibody titers and shed more virus. This knowledge is important for the management of FCoV infections in multi-cat environments, but the results indicate that antibody measurement cannot replace fecal RT-qPCR.

## 1. Introduction

Feline coronavirus (FCoV) is a viral pathogen infecting cats worldwide. It is highly contagious [1]: nearly 100% of cats become infected when exposed, usually horizontally via the fecal-oral route [2,3]. Additionally, the virus can persist in excretions in the environment for up to several weeks [4], rendering indirect transmission, e.g., via caregivers, a possible route of infection. The prevalence of FCoV in the cat population is very high; this is especially true for multi-cat households, in which prevalence can be as high as 90% [5,6,7]. Only about 7–14% of FCoV-infected cats go on to develop feline infectious peritonitis (FIP) [8], which arises from the mutation of the avirulent enteric FCoV (FECV) with a tropism for the gastrointestinal tract to the highly virulent FIP virus (FIPV), with a tropism for monocytes/macrophages within an infected cat [9,10]. The pathological hallmark of FIP is a granulomatous vasculitis and perivasculitis [11], which develops as the result of an immunological process. Subsequent to the infection of monocytes/macrophages and replication of FCoV within these cells, multiple cytokines, including tumor necrosis factor alpha, and adhesion molecules, are produced and ultimately lead to increased vascular permeability and the development of pyogranulomas [11,12,13,14,15,16]. Both healthy FCoV-infected cats and cats suffering from FIP can shed FCoV in their feces [17,18,19]. Fecal shedding of mutated FCoV in cats with FIP is rare, and if it occurs, this virus is not infectious for other cats [20].

If a cat becomes infected with FCoV, fecal virus shedding usually begins within one week after infection [3,21], and in general follows three possible patterns: some cats become chronic FCoV carriers, and persistently, even in the absence of re-infection, shed the virus for varying times or even lifelong; some cats will eliminate the infection and stop shedding FCoV, but can become re-infected; and consequently, the majority of cats shed FCoV intermittently or (more likely) continuously become re-infected throughout their lives [3,22,23]. It has been shown that young and immunosuppressed cats shed more virus with their feces than older or healthy cats [3]. Cats infected with feline immunodeficiency virus, for instance, shed 10–100× more FCoV than healthy age-matched controls [9].

After experimental infection with FCoV, antibodies first appear in the blood after about one to three weeks [21,23,24]. Antibodies are not effective to clear enteric viral colonization and to prevent re-infection. Additionally, antibodies do not preclude the development of FIP. In fact, experimental studies even demonstrated a more rapid and severe course of the disease in cats with pre-existing antibodies, due to antibody-dependent enhancement [25,26,27,28], although this phenomenon most likely does not occur under field conditions [22]. Moreover, FCoV-infected monocytes/macrophages do not present viral antigen on their cell surface, thereby avoiding antibody-mediated lysis by the immune system [29]. Previous studies have proposed a correlation between the antibody titer of a cat and its fecal FCoV shedding. These studies have found that cats with higher antibody titers are more likely to be FCoV shedders, whereas cats with lower antibody titers are less likely to shed virus in their feces and most cats without antibodies do not shed FCoV [3,30,31]. However, the results are controversial, as some studies did not confirm these findings [32,33,34]. Additionally, it has been reported that not all cats with antibodies are virus shedders [32]; only approximately 30% antibody-positive cats shed FCoV in their feces [35]. Similarly, there are reports of some antibody-negative cats that shed FCoV in their feces [18,31]. There is only one dissertation, which was not published in a peer-reviewed journal, suggesting a correlation between the frequency and the amount of fecal FCoV shedding, and between the antibody titer and the amount of FCoV shedding [36].

For many years, FIP was considered a uniformly fatal disease. Very recently, however, novel drugs, such as a nucleoside analog, GS-441524, were shown to have antiviral efficacy in cats with experimental and natural FIP and in cats with asymptomatic FCoV infection [37,38,39,40]. Nevertheless, these medications are not yet commercially available and might not be affordable for all cat owners, especially for cat breeders and rescue shelters. Additionally, there are serious concerns that its use to eliminate FCoV infection rather than to treat FIP might give rise to resistant viral mutants. Therefore, the eradication of FCoV infection in multi-cat households, if at all possible, still widely relies on hygiene and husbandry measures, the identification of virus carriers and separation of shedders from non-infected cats [33]. Although this can be achieved by testing repeat fecal samples for FCoV RNA by quantitative reverse transcriptase polymerase chain reaction (RT-qPCR), such a procedure will prolong the period in which the shedding status of a cat is unknown [41], since the collection of sequential fecal samples and analysis by RT-qPCR will take much longer than antibody measurement from one blood sample. Therefore, an attempt to quickly detect antibodies as an indication of the amount of shedding has been proposed.

It was the aim of this study to determine a possible correlation between the FCoV antibody titer and fecal FCoV shedding in catteries. The questions addressed included (1) whether cats with higher antibody titers would shed higher concentrations of FCoV with their feces; and (2) whether cats shed FCoV more frequently throughout the study period and (3) whether cats shedding FCoV in all four fecal samples collected would have higher antibody titers and shed higher concentrations of FCoV than cats that shed virus only intermittently. Indeed, the results obtained suggest that in cats from German catteries, the antibody titer is correlated with mean fecal virus load. Cats with antibodies were more likely to be FCoV shedders than non-shedders. Cats with higher antibody titers shed FCoV more frequently and with a higher mean fecal virus load than cats with lower FCoV antibody titers or without antibodies. Cats shedding FCoV in all four fecal samples had higher FCoV antibody titers and shed higher concentrations of FCoV than cats shedding FCoV intermittently. However, there were nine cats without antibodies that shed FCoV. Thus, antibody testing cannot replace fecal testing by RT-qPCR.

## 2. Results

**Figure 1:**
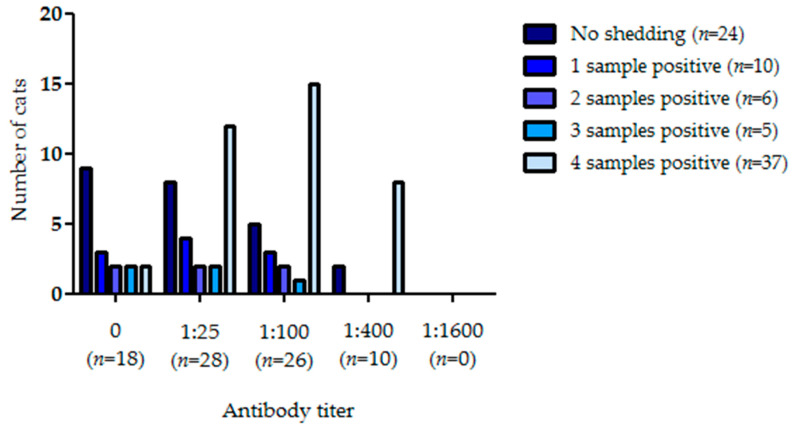
Antibody titers of cats not shedding feline coronavirus (FCoV) and of cats with one, two, three, or four fecal samples positive for FCoV RNA by quantitative reverse transcriptase polymerase chain reaction (RT-qPCR). Fecal samples with weak positive RT-qPCR results were considered positive; samples below the limit of quantification were considered negative.

Anti-FCoV antibodies were detected in 64 of the 82 cats (78%) included in the study (Figure 1). All 19 catteries homed at least one cat with antibodies. FCoV RNA could be detected in at least one of the four fecal samples collected at intervals of 5-30 days by RT-qPCR in 58 of the 82 included cats (71%), and all 19 catteries homed at least one cat that was shedding FCoV in at least one fecal sample.

Of the 18 cats without detectable antibodies, nine cats (50%) shed FCoV at least once. Of the cats with antibodies, 20/28 (71%) with an antibody titer of 1:25, 21/26 (81%) with a titer of 1:100, and 8/10 (80%) with a titer of 1:400 shed FCoV at least once. None of the cats had an antibody titer of 1:1600. Most (71%) of the 24 cats that were not shedding FCoV either did not have antibodies (9/24; 38%) or only a very low titer of 1:25 (8/24; 33%). Nevertheless, 7/24 (29%) cats that were RT-qPCR-negative in all four fecal samples had an antibody titer of at least 1:100. 

### 2.1. Correlation between Mean Fecal FCoV Load and Antibody Titer

Fecal virus load ranged from 2.2 × 10^6^ to 4.5 × 10^12^ per gram (g) of feces (median 6.8 × 10^9^ per g feces) in cats shedding FCoV (Table 1). Cats with higher anti-FCoV antibody titers also had significantly higher mean fecal virus loads. There was a weak positive correlation between the antibody titer and the calculated mean fecal virus load (mean of all four fecal samples) (Spearman *r* = 0.2984, 95% confidence interval 0.07761–0.4913; *p* = 0.0072).

### 2.2. Correlation between Frequency of Fecal FCoV Shedding and Antibody Titer and Mean Fecal FCoV Load

In addition to shedding larger amounts of FCoV with their feces, cats with higher antibody titers also shed FCoV more frequently. Antibody titers were significantly higher in cats with more frequent than in cats with less frequent FCoV shedding (*p* = 0.0063; Figure 1). However, when comparing the cats in different groups (based on their frequency of FCoV shedding), this difference was only significant between cats that were RT-qPCR-negative in all four fecal samples and cats that were RT-qPCR-positive in all four fecal samples (Dunn’s test; *p* < 0.05). Two cats that were RT-qPCR-positive in all four fecal samples did not have antibodies and two cats that were RT-qPCR-negative in all four fecal samples had a high antibody titer of 1:400.

When considering the mean fecal virus load and frequency of FCoV shedding, there was a slight tendency that cats shedding FCoV more frequently shed higher concentrations of FCoV RNA, but that association was not significant (*p* = 0.1323; Table 2).

### 2.3. Correlation between FCoV Shedding in All Four vs. One to Three Samples and Antibody Titer

Fifty-eight of the 82 cats (71%) were positive for FCoV RNA by RT-qPCR in at least one of the four fecal samples collected. Of those 58 cats, 37 (64%) were RT-qPCR-positive in all four fecal samples. These 37 cats shedding in all four fecal samples had significantly higher antibody titers than cats that were RT-qPCR-positive in only one, two, or three fecal samples (*p* = 0.0014; Table 3).

**Figure 2:**
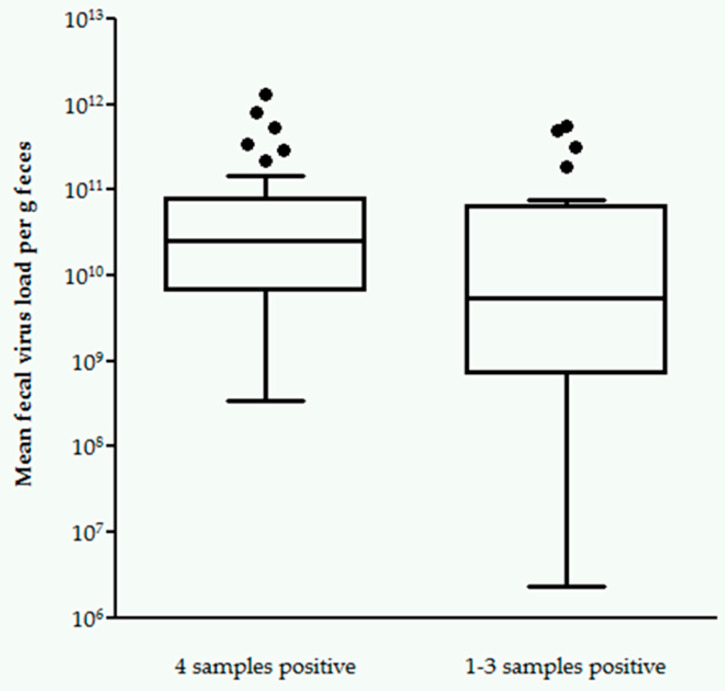
Box and whisker plot. Comparison of cats with one to three versus four fecal samples positive for feline coronavirus (FCoV) RNA by quantitative reverse transcriptase polymerase chain reaction (RT-qPCR), with cats with four fecal samples positive FCoV RNA by RT-qPCR. Mean fecal virus load per gram (g) of feces of cats with four RT-qPCR-positive samples was significantly higher compared to cats with only one, two, or three RT-qPCR-positive samples (*p* = 0.0475). Mean fecal FCoV load was calculated as the mean of all four fecal samples from each cat. Fecal samples with weak positive RT-qPCR results were considered positive; samples below the limit of quantification were considered negative. The two cats with only one weak RT-qPCR-positive or one sample below the limit of quantification and three RT-qPCR-negative fecal samples each were excluded from analysis.

Moreover, when comparing shedders with four RT-qPCR-positive fecal samples and shedders with one to three RT-qPCR-positive fecal samples, shedders with four RT-qPCR-positive fecal samples shed significantly higher mean fecal virus loads (3.45 × 10^8^–1.31 × 10^12^ viral copies per g feces, median 2.53 × 10^10^; *p* = 0.0475; Figure 2) than shedders with one to three RT-qPCR-positive fecal samples (2.27 × 10^6^–5.50 × 10^11^ viral copies per g feces, median 5.47 × 10^9^).

## 3. Discussion

The aim of this study was to investigate a possible correlation between FCoV antibody titer and fecal virus shedding in healthy cats living in German catteries. The majority of antibody-positive cats in this study (49/64, 77%) shed FCoV in their feces. However, the study also showed that this assumption is not absolute, since 9/18 (50%) of cats without antibodies also were FCoV shedders and 2/10 (20%) of the cats with a high antibody titer of 1:400 did not shed FCoV. Previously, only 30% antibody-positive cats were reported to shed FCoV [30]. A possible reason for this disagreement is the way in which shedding was confirmed in the two studies. In the present study, FCoV RNA was detected in fecal samples by RT-qPCR. This technique is very sensitive. In the previously published study, RT-PCR was not yet available, so the classification of a cat as shedder occurred via its infectivity to other cats. More precisely, kittens of antibody-positive queens were monitored for the development of antibodies and it was shown that 38% of the litters developed antibodies, indicating viral shedding occurred in around 30% of the queens [30]. Of course, this approach is not directly comparable to the testing of feces by molecular methods such as RT-qPCR, as performed in the present study.

According to previous studies, FCoV antibody titers are significantly higher in cats that shed FCoV with their feces than in cats that do not shed FCoV [3,30,31]. Additionally, it was proposed that cats without FCoV antibodies are not shedding FCoV and that, as a consequence, it would be safe to introduce an antibody-negative cat into a FCoV-free household [3,30,31]. However, a smaller number of studies reported discordant results, refuting a correlation between FCoV antibody titer and the likelihood of FCoV shedding [32,33,34]. Pedersen et al. [3] tried to explain this discrepancy by different ways of data evaluation. Correlation between antibody titer and fecal virus shedding was significant when shedding and non-shedding cats were looked at as groups. Conversely, there was a substantial overlap when evaluating individual cats [3]. This, at least in part, is also true for the cats in the present study. Most cats with a high antibody titer of 1:400 were shedding FCoV (and shed FCoV in all four fecal samples), but two cats with a titer of 1:400 did not shed FCoV in any of the four fecal samples. Even more discrepancy could be seen among the cats without antibodies: 9/18 of the cats without antibodies shed FCoV at least once during the study period. This is a result that, despite the initial belief that antibody-negative cats would never shed FCoV [30,31,32], has been reported in the literature before [18,34]. It is possible that the contamination of fecal samples by other FCoV-positive cats had occurred in the same multi-cat household. In order to avoid contamination, rectal swabs could be used for testing purposes by cat breeders [31], but this would reduce the amount of available fecal material. Contamination of samples in the laboratory cannot fully be excluded, but seems rather unlikely given that extensive quality controls were included in the RT-qPCR protocol. More likely, it is conceivable that serum/plasma samples were obtained early in an infection, at a time before the cats developed antibodies, but at which they already shed FCoV with their feces. Shedding of FCoV before the time of antibody development has already been reported in kittens [34]. Since serial antibody measurements were not performed in the present study, such scenario cannot be confirmed. Alternatively, the documentation of fecal FCoV shedding in antibody-negative cats could be explained by localized infection. It is known that FCoV replication can stay confined to the gastrointestinal tract in some FCoV-infected cats, resulting in fecal FCoV shedding without the development of antibodies [18]. This might also have occurred in the cats in the present study.

Antibody testing and the separation of antibody-negative cats in the past has been suggested as method to clear FCoV infection from a breeding cattery [42] or prevent the introduction of FCoV infection into the FCoV-free geographical area of the Falkland Islands [43]. However, as clearly shown by the results of the present study, although the antibody titer can give an idea on a cat’s shedding status, a negative antibody titer in a cat does not exclude fecal shedding. Thus, the introduction of cats into FCoV-free environments on the basis of a negative antibody test still bears the risk of introducing a FCoV-shedding cat. On the other hand, the separation of all antibody-positive cats might not be necessary, because not all cats with antibodies shed FCoV with their feces.

The next question to be answered was whether cats with higher antibody titers also shed higher concentrations of FCoV RNA with their feces. In this study, there was a weak positive correlation between the quantity of antibodies and the mean fecal virus load determined by RT-qPCR, indicating that cats with higher antibody titers were more likely to shed FCoV more intensely compared to cats with low antibody titers and cats without antibodies. It is possible that a higher amount of viral replication, as demonstrated by a higher fecal virus load, also leads to increased antibody production, and thus, higher antibody titers. However, since there is some overlap, this correlation is only weak, demonstrating that antibody measurement alone is not sufficient to differentiate high intensity from low intensity shedders. So far, there is only one report, which was not published in a peer-reviewed journal, that also suggested a correlation between FCoV antibody titer and the intensity of fecal virus shedding [36]. Clarification of such information, however, is of practical importance, since especially high intensity shedders are a concern in environments where many cats are housed together in a limited space. These cats shedding large amounts of FCoV pose a high risk of transmission to non-infected cats, and in order to reduce infection pressure in multi-cat environments, the contact of non-infected cats to litter trays of high intensity shedders must be avoided [33,44]. However, the routine practice of testing four sequential fecal samples or rectal swabs taken one week to one month apart for FCoV by RT-qPCR, while increasing the likelihood of correctly identifying non-shedders, leads to a prolonged period of time, in which the shedding status of cats in the multi-cat environment is unknown [31,41]. This is especially problematic in rescue shelters, where new cats with unknown FCoV status are to be introduced. Antibody titers (for which only one blood sample is necessary), in contrast, are available much faster and therefore could help to distinguish high intensity from low intensity shedders or cats not shedding FCoV. The results of the present study indicate that the risk of shedding large amounts of FCoV will increase with the level of a cat’s antibody titer, and a cat without antibodies most likely will not shed large amounts of FCoV. This understanding might help to at least estimate whether strict quarantine measures are indispensable or not for each individual cat. The practice of separating high intensity shedders, low intensity shedders and non-shedders in a cat population in order to eliminate FCoV infection is being proposed, but not without controversy [44,45]. It has been suggested that antibody measurement might be of importance in breeding catteries, in which kittens might be initially protected from infection by maternally-derived antibodies (MDA), that are transferred by nursing from antibody-positive queens, but can become infected as soon as MDA wane. Isolation of pregnant queens before birth and early weaning of kittens at an age of five to six weeks, before the loss of MDA, has been proposed [30,33]. However, this protocol has been questioned, since the adequate socialization of isolated kittens is a concern and FCoV infection can occur as early as two weeks of age [46].

After fecal-oral FCoV infection, three possible shedding patterns have been observed. Cats can shed FCoV intermittently [3,22,23,31,32,47]. Most likely, this is only partially caused by intermittent fecal excretion, but also by re-infection with either the same or a different FCoV strain throughout their lives [22]. Some cats will shed FCoV for weeks or months and eventually cease fecal shedding. Up to 13% of cats will shed FCoV persistently for a prolonged period of time and sometimes even lifelong [3,22,31,32,47]. The exact percentage of cats following each pattern is unknown and likely varies depending on the epidemiological situation and virulence of the infecting FCoV strain. In order to correctly characterize a FCoV-shedding cat for eradication purposes, sequential fecal samples must be obtained and four fecal samples, collected 5–30 days apart, were obtained from each cat in this study. Results indicate that cats had significantly higher antibody titers if they shed FCoV more frequently. When considering the results of the individual cats, it becomes apparent that most cats (71%) that did not shed FCoV in any fecal sample either did not have antibodies (9/24) or only had a low antibody titer of 1:25 (8/24), whereas most cats with a high antibody titer of 1:400 (8/10; 80%) shed FCoV in all four samples. Nevertheless, although there was a tendency for cats shedding FCoV more frequently (possibly due to continuous re-infection) to have higher antibody titers, again there was some overlap, and the correlation was only significant for cats shedding in all four fecal samples compared to non-shedders.

It was also determined whether cats shedding FCoV in all four samples had higher antibody titers than cats shedding FCoV in only one, two, or three fecal samples. The latter cats probably eliminated the infection at some point, shed intermittently or became re-infected. In this context, it is of special interest to evaluate whether measurement of the antibody titer can indicate if a cat likely is continuously shedding FCoV, and thus, poses a permanent risk of infection to other cats. It could be shown that, in this population, cats that were RT-qPCR-positive in all four samples had significantly higher antibody titers than cats, that were RT-qPCR-positive only intermittently in one, two, or three fecal samples. Additionally, cats shedding FCoV in all four samples also had significantly higher mean fecal virus loads than cats shedding only intermittently. This confirms the results of one study performed as part of a doctoral thesis, which followed 77 FCoV-infected cats over a period of 24 weeks, and could demonstrate that the amount of FCoV RNA shed was significantly higher with higher shedding frequency (defined as % of RT-qPCR-positive fecal samples) [36].

Twenty-four cats were RT-qPCR-negative in all four fecal samples, indicating that they persistently did not shed FCoV. These cats either did not have contact to FCoV, which seems rather unlikely given the fact that all of the included catteries harbored at least one FCoV-shedding cat and the virus is highly contagious [7,23,33]. Alternatively, these cats could have been resistant to FCoV. Resistant cats never shed FCoV and do not develop FCoV antibodies or only very low titers [31]. The mechanism for this is unknown [46]. Interestingly, however, 15 of the 24 non-shedders in this study did have antibodies. Mostly, these cats had low antibody titers. A possible explanation for this could be that the cats had been infected previously, but ceased shedding before the beginning of the study and antibodies persisted for a longer period of time. The longest period documented in the literature for which a cat remained antibody-positive after it stopped FCoV shedding was 25 months and decreasing antibody titers were demonstrated in studies following cats ceasing to shed FCoV over time [31]. It is possible that antibody titers were in the process of declining in the cats in the present study as well.

This study had some limitations. First, blood samples were obtained from each cat at only one time point. Therefore, it was impossible to follow the cats and the development of their antibody titers over time. This would have been especially interesting in cats that ceased to shed virus or in persistent viral shedders. A second limitation is the fact that most cats were housed very closely, and each cattery housed at least one cat that shed FCoV. Thus, cross-contamination between the individual cats cannot fully be excluded, even though cat breeders acted with great caution to separate fecal samples. Thirdly, in the past, the term chronic FCoV carrier has been used for cats shedding FCoV for at least nine months [31]. Cats in the present study were followed for a maximum of four months. Therefore, it is not known whether cats shedding FCoV in all four fecal samples were true FCoV carriers or might have ceased shedding after the end of the study period.

## 4. Materials and Methods

The study was performed prospectively and included 82 cats originating from 19 German catteries. Catteries were distributed all over Germany. A cattery was defined as a cat household with at least five cats and at least one intact queen for breeding. Breeders collected samples from a variable non-predefined number of cats in their cattery. Breeds included British Shorthair (*n* = 24), Bengal (*n* = 19), Birman (*n* = 15), Maine Coon (*n* = 7), Scottish Fold (*n* = 7), Norwegian Forest (*n* = 5), Turkish Van (*n* = 3), and Turkish Angora (*n* = 2). The study protocol was approved by the responsible veterinary authority (Regierung von Oberbayern; reference number 55.2-1-54-2532.2-14-13).

Cat breeders collected four fecal samples from the cats, at varying time intervals from 5–30 days between collections. Samples were frozen at −20 ℃ until examination. All fecal samples were examined for FCoV load per g of feces by RT-qPCR. A mean fecal virus load was calculated for each cat from all RT-qPCR-positive samples. For RT-qPCR, total nucleic acid was extracted from fecal samples by QIAamp DNA Blood BioRobot MDx Kit on an automated Qiagen platform (QIAGEN GmbH, Hilden, Germany) according to the manufacturer instructions, with slight modifications. The RT-qPCR and total nucleic acid extraction procedures were adapted from previously published protocols [24,48]. A quantitative real-time PCR based on the 7b gene [48] was performed as a singleplex reaction at a commercial reference laboratory (IDEXX Laboratories, Ludwigsburg, Germany). Real-time PCR was run with six quality controls, including: (1) PCR-positive controls (quantitatively;), using synthetic DNA covering the real-time PCR target region (Integrated DNA Technologies IDT, Coralville, IA, USA), (2) PCR-negative controls (PCR-grade nuclease free water), (3) negative extraction controls (extraction positions filled with lysis solution and PCR-grade nuclease free water only), (4) RNA pre-analytical internal sample control targeting feline ssr rRNA (18S rRNA) gene complex, (5) a swab-based environmental contamination monitoring control, and (6) spike-in internal positive control (using lambda phage DNA). These controls assessed the functionality of the PCR test protocols (1) for the absence of contamination in the reagents (2) and laboratory (5), the absence of cross-contamination during the extraction process (3), quality and integrity of the RNA as a measure of sample quality (4), reverse transcription protocol (5 and 6), and the absence of PCR-inhibitory substances as a carryover from the sample matrix (6).

The interpretation of RT-qPCR results is demonstrated in Table 4. If RT-qPCR was initially weak positive (threshold cycle (Ct) > 40), RT-qPCR was repeated in duplicate. Depending on the results of this duplicate repetitive analysis, results were then categorized (Table 4). Questionable RT-qPCR results occurred in four cats. The first cat had one fecal sample that was weak positive by RT-qPCR (low concentrations of FCoV RNA were detected but viral load was below the limit of quantification); the other three fecal samples of this cat were RT-qPCR-negative. The second cat had two RT-qPCR-negative, one RT-qPCR-positive and one weak positive fecal sample. The third cat had three RT-qPCR-positive and one fecal sample below the limit of quantification. The fourth cat had three RT-qPCR-negative and one fecal sample below the limit of quantification. Weak positive samples were considered positive for further analysis. Samples below the limit of quantification were considered negative for further analysis. The two cats with only one weak RT-qPCR-positive or one below the limit of quantification and three RT-qPCR-negative fecal samples each were excluded from all analyses involving fecal virus load, since mean fecal virus load could not be determined.

Additionally, one serum and/or plasma sample was obtained from each of the 82 cats, around the time of collection of the last fecal sample of each cat. Only a serum sample was available from 68 cats, only a plasma sample was available from 10 cats, and both a serum and a plasma sample were available from four cats. Serum and/or plasma antibody titers were determined by an indirect immunofluorescence assay as previously described [49,50], using PD-5 cells (swine origin) infected with transmissible gastroenteritis virus (Perdue strain). Infected cells were mixed with uninfected cells; the latter served as internal negative control. Cat samples were tested at dilutions of 1:25, 1:100, 1:400 and 1:1600. The fluorescein isothiocyanate (FITC) conjugated secondary antibody (rabbit anti cat IgG (H + L), Nordic-MUbio, Sustern, Netherlands; LuBio Sience GmBH, Luzern, Switzerland) was diluted at 1:40. The serum samples and the conjugate were each incubated at 37 °C for 1 h in a humid chamber, followed by three phosphate buffered saline (PBS) wash steps, and drying of the slide surface with absorbing paper, preventing the wells from drying out. A positive control (aliquoted serum sample of a FCoV-antibody-positive field cat) and a negative control (aliquoted serum from a specified pathogen free FCoV-antibody-negative cat) were run with each slide. The slides were covered with a cover slide, fixed with a few drops of drops of PBS-Glycerol (1:3) and read using a fluorescence microscope (Leica DMLB, Leica Microsystems, Heerbrugg, Switzerland). The antigen preparations used to prepare the slides were tested for the absence of contaminating viruses by RT-PCR and PCR, as previously described [51].

The correlation between level of antibody titer and mean fecal virus load was determined by Spearman’s correlation. Kruskal–Wallis test including Dunn’s post-test was performed in order to determine a possible correlation between antibody titer and the frequency of virus shedding in an individual cat. Kruskal–Wallis test was also performed to evaluate a possible correlation between frequency and amount of fecal virus shedding. Mann–Whitney U test was applied when comparing cats shedding in all four fecal samples collected and cats shedding in only one to three fecal samples, in order to determine a possible correlation between the frequency of FCoV shedding and antibody titer and between the frequency of FCoV shedding and mean fecal virus load. Statistical analysis was performed using Graph Pad Prism version 5.04 (GraphPad Software, San Diego, CA, USA).

## 5. Conclusions

In this study, cats with antibodies and especially cats with higher antibody titers were more likely to shed FCoV; additionally, they shed FCoV significantly more frequently throughout the study period and had significantly higher mean fecal virus loads than cats with lower antibody titers or without antibodies. Cats shedding FCoV in all four fecal samples collected had significantly higher antibody titers and significantly higher mean fecal virus loads than cats shedding FCoV intermittently. However, it is important to note that even antibody-negative cats shed FCoV, and only 77% of the antibody-positive cats shed FCoV with their feces. Therefore, the measurement of the antibody titer can help in managing FCoV infection in catteries or multi-cat households, but cannot replace the examination of fecal samples by RT-qPCR. RT-qPCR and antibody titer taken together can give a more reliable picture of the status of an individual cat than each test alone.

## Figures and Tables

**Table 1:** Fecal feline coronavirus (FCoV) load per gram of feces of cats with different antibody titers detected by quantitative reverse transcriptase polymerase chain reaction (RT-qPCR). Fecal FCoV load was calculated as the mean of all four fecal samples from each cat. The two cats with only one weak RT-qPCR-positive or one sample below the limit of quantification and three RT-qPCR-negative fecal samples each were excluded from analysis.

Antibody Titer	Number of Cats	Fecal FCoV Load
**Negative**	8	2.2 × 10^6^–5.0 × 10^11^ (median 2.6 × 10^9^)
**1:25**	20	6.4 × 10^6^–8.0 × 10^11^ (median 1.3 × 10^10^)
**1:100**	21	7.4 × 10^8^–1.3 × 10^12^ (median 1.2 × 10^10^)
**1:400**	8	3.4 × 10^8^–2.9 × 10^11^ (median 2.0 × 10^10^)
**1:1600**	0	n. a.

n. a.: not applicable.

**Table 2:** Mean fecal feline coronavirus (FCoV) load per gram of feces of cats with different frequencies of FCoV shedding detected by quantitative reverse transcriptase polymerase chain reaction (RT-qPCR). Fecal FCoV load was calculated as the mean of all four fecal samples from each cat. Fecal samples with weak positive RT-qPCR results were considered positive; samples below the limit of quantification were considered negative. The two cats with only one weak RT-qPCR-positive or one sample below the limit of quantification and three RT-qPCR-negative fecal samples each were excluded from analysis.

Shedding Frequency	Number of Cats	Fecal FCoV Load
One sample RT-qPCR-positive	9	2.2 × 10^6^–3.1 × 10^11^ (median 5.2 × 10^9^)
Two samples RT-qPCR-positive	6	5.1 × 10^8^–4.9 × 10^11^ (median 3.7 × 10^9^)
Three samples RT-qPCR-positive	5	2.5 × 10^9^–5.5 × 10^11^ (median 1.1 × 10^10^)
Four samples RT-qPCR-positive	37	3.4 × 10^8^–1.3 × 10^12^ (median 2.5 × 10^10^)

**Table 3:** Numbers of cats with different antibody titers with positive quantitative reverse transcriptase polymerase chain reaction (RT-qPCR) detecting feline coronavirus (FCoV) RNA in all four (continuous shedders) or only one to three (non-continuous shedders) fecal samples. Fecal samples with weak positive RT-qPCR results were considered positive; samples below the limit of quantification were considered negative.

Antibody Titer	Continuous Shedders (% of total)	Non-Continuous Shedders (% of total)	Total Shedding Cats
**Negative**	2 (22%)	7 (78%)	**9**
**1:25**	12 (63%)	7 (37%)	**19**
**1:100**	15 (68%)	7 (32%)	**22**
**1:400**	8 (100%)	0 (0%)	**8**
**Total**	**37 (64%)**	**21 (36%)**	**58**

**Table 4:** Interpretation of results of the quantitative reverse transcriptase polymerase chain reaction (RT-qPCR).

**Ct Value**	**Result**
<40	Positive
>40	Weak positive (RT-qPCR had to be repeated in duplicate)
No Ct	Negative
**Ct Value in Duplicate Repetitive Analysis**	**Result**
1× < 40 and 1× > 40	Positive
2× > 40	Weak positive ^1^
1× < 40 and 1× no Ct	Questionable positive
1× > 40 and 1× no Ct	Below the limit of quantification
2× no Ct	Negative

Ct, threshold cycle; ^1^ Low concentration of feline coronavirus RNA detected.
